# Common alleles contribute to schizophrenia in CNV carriers

**DOI:** 10.1038/mp.2015.143

**Published:** 2015-09-22

**Authors:** K E Tansey, E Rees, D E Linden, S Ripke, K D Chambert, J L Moran, S A McCarroll, P Holmans, G Kirov, J Walters, M J Owen, M C O'Donovan

**Affiliations:** 1 Medical Research Council Centre for Neuropsychiatric Genetics and Genomics, Institute of Psychological Medicine and Clinical Neurosciences, School of Medicine, Cardiff University, Cardiff, UK; 2 Department of Psychiatry and Psychotherapy, Charité-Universitätsmedizin Berlin, Berlin, Germany; 3 Stanley Center for Psychiatric Research, Broad Institute of MIT and Harvard, Cambridge, MA, USA; 4 Department of Genetics, Harvard Medical School, Boston, MA, USA

## Abstract

The genetic architecture of schizophrenia is complex, involving risk alleles ranging from common alleles of weak effect to rare alleles of large effect, the best exemplar of the latter being large copy number variants (CNVs). It is currently unknown whether pathophysiology in those with defined rare mutations overlaps with that in other individuals with the disorder who do not share the same rare mutation. Under an extreme heterogeneity model, carriers of specific high-penetrance mutations form distinct subgroups. In contrast, under a polygenic threshold model, high-penetrance rare allele carriers possess many risk factors, of which the rare allele is the only one, albeit an important, factor. Under the latter model, cases with rare mutations can be expected to share some common risk alleles, and therefore pathophysiological mechanisms, with cases without the same mutation. Here we show that, compared with controls, individuals with schizophrenia who have known pathogenic CNVs carry an excess burden of common risk alleles (*P*=2.25 × 10^−17^) defined from a genome-wide association study largely based on individuals without known CNVs. Our finding is not consistent with an extreme heterogeneity model for CNV carriers, but does offer support for the polygenic threshold model of schizophrenia. That this is so provides support for the notion that studies aiming to model the effects of rare variation may uncover pathophysiological mechanisms of relevance to those with the disorder more widely.

## Introduction

Genetic risk for schizophrenia arises from alleles across the entire frequency spectrum.^1, 2, 3, 4, 5^ With respect to common alleles, a recent genome-wide association study (GWAS) reported 108 loci that influence risk for schizophrenia; although each locus contributes only a small effect on risk (typical odds ratios (ORs) <1.1), it has been estimated that, ultimately, between a third and a half of the genetic risk can be attributed to common alleles.^1, 6, 7, 8^ Recent exome-wide sequencing studies have also confirmed a role in schizophrenia for rare single-nucleotide variants,^2, 3^ although, to date, the only specific rare variants that have been robustly implicated in the disorder are of the mutation class known as copy number variants (CNVs).

CNVs are large, but submicroscopic, chromosomal insertions and deletions. Currently, around 11 recurrent CNVs have been convincingly associated with schizophrenia.^4, 5, 9, 10, 11, 12, 13^ Unlike common alleles, CNVs, at least those that have been identified, confer large effects on risk (OR ranging from about 2 to 60).^4, 5, 9, 10, 11, 12, 13^ Nevertheless, they are not completely penetrant,^14, 15^ that is, they are not sufficient to cause schizophrenia. They also lack diagnostic specificity as the same CNVs are known to influence the risk for several neuropsychiatric disorders, including intellectual disability, autism spectrum disorders and attention-deficit hyperactivity disorder, as well as disorders in other systems, including cardiovascular, renal, immune and musculoskeletal.^10, 14, 16^

Competing hypotheses have been proposed about the genetic architecture and influence of CNVs, as well as other types of rare mutations, on schizophrenia. At the one end of the spectrum, an extreme heterogeneity model proposes that schizophrenia represents a highly disparate set of distinct disorders, among which those with a specific mutation form homogeneous but small subgroups. This model, also known as a multiple rare variant model,^17^ is no longer generally sustainable given the success in identifying common risk variants, but it may apply to the subset of cases carrying rare high-penetrance mutations such as CNVs. That all known CNVs have incomplete penetrance for schizophrenia suggests that even under this model, their clinical effects depend on other risk factors, genetic and/or environmental.^15, 18^ If the hypothesis of extreme heterogeneity extends from distinct alleles to distinct pathophysiology, these additional risk factors should not overlap between carriers of different CNVs, or between carriers of CNVs and people with schizophrenia who do not carry CNVs. Examples of additional CNV-specific risk factors that have been considered include, for example, a ‘second-hit' somatic or germ-line mutation occurring at a gene within the CNV region but on the unaffected chromosome, a second hit mutation elsewhere in the genome, or mutation-specific environmental exposures. If this is true, the results obtained from studies that seek to model the effects of CNVs and other types of rare mutation may be useful for understanding pathological mechanisms of disorder in those individuals, but tell us little about the generality of cases with a diagnosis of schizophrenia.

At the other end of the spectrum, schizophrenia can be considered a polygenic/multifactorial disorder where disorder is the result of an accumulation of risk factors sufficient to surpass a threshold of disease liability.^19, 20, 21, 22, 23^ Given the existence of a sufficiently large number of risk alleles segregating in the population, it may be that each individual case has a unique risk profile, in some cases including a CNV or other type of rare mutation. Nevertheless, cases will also carry risk alleles that are shared with many others with the disorder, making the concept of extreme heterogeneity at the pathophysiological level largely meaningless.

Here, we test these extreme models by evaluating whether individuals with a diagnosis of schizophrenia who carry a schizophrenia-associated CNV also share a common risk allele burden with those who have schizophrenia but no schizophrenia-associated CNV. To measure the common risk allele burden, we use the polygenic risk profile score (RPS) method that has been widely shown to be a powerful and valid tool for this purpose and has, for example, revealed common allele burden overlaps between schizophrenia and bipolar disorder.^1, 9, 24^ Essentially, an individual's RPS reflects the total number of independent risk alleles (weighted by their effect sizes) carried by that individual, a risk allele being defined as one that surpasses specified thresholds of significance (*P*_T_) for disease association in an independent GWAS study (that is, one that does not contain that individual). In the present study, we define schizophrenia susceptibility alleles using the largest published GWAS of schizophrenia.^1^ Under the polygenic liability threshold model, but not the heterogeneity model, we predict that CNV carriers with schizophrenia will have an increased burden (RPS) of the common risk alleles defined from the wider population of people with schizophrenia.

## Materials and methods

### Samples

The CLOZUK schizophrenia sample used here has been described elsewhere.^1^ Ascertainment of samples has been described previously.^1^ Briefly, cases were ascertained with the assistance of Novartis, the manufacturer of a proprietary form of clozapine (Clozaril). The sample consisted of individuals with treatment-resistant schizophrenia according to the clozapine registration forms completed by treating psychiatrists. The controls were from Wellcome Trust Case Control Consortium 2 (http://www.wtccc.org.uk/info/access_to_data_samples.html). Additional 900 controls were recruited from the UK National Blood Transfusion Service by Cardiff University. CLOZUK samples used in this analysis include only those individuals who passed both genome-wide association quality control and CNV quality control resulting in a sample of 6005 controls and 5423 cases. The UK Multicentre Research Ethics Committee approved the study and all control samples were from participants who provided informed consent.

Although the CLOZUK is a clinical rather than a research-diagnosed sample, we^24^ and subsequently the Psychiatric Genomics Consortium^1^ have shown that the sample is valid for genetic studies of the disorder. Specifically of relevance to the present study, the CLOZUK sample is indistinguishable from other samples obtained using research diagnoses with respect to common genetic risk factors^1, 24^ and to the rate of occurrence or specific type of CNVs.^5^

### Genome-wide genotype data

Samples were genotyped using the Illumina HumanOmniExpress-12v1, HumanOmniExpressExome 8v1 and Human 1.2M Duo custom BeadChips v1 (Illumina Inc., San Diego, CA, USA). As part of the Schizophrenia Working Group of the Psychiatric Genomics Consortium (PGC-SCZ) analytic pipeline,^1^ samples underwent routine quality control using PLINK^25^ and were imputed using the prephasing/imputation stepwise approach implemented in IMPUTE2/SHAPEIT^26, 27^ and 1000Genomes (August 2012, release 'v3.macGT1') as the reference data set. Only SNPs with high confidence (imputation information score>0.9) were used in this analysis.

### CNV calling

CNVs were called using PennCNV^28^ following standard protocols and adjusting for GC content. Samples were excluded if for any one of the following standard quality control statistics they constituted an outlier: log *R* ratio standard deviation, B-allele frequency drift, wave factor and total number of CNVs. Quality control for CNVs included joining CNVs called in the same individual if the distance separating them was less than 50% of their combined length. The resulting CNVs were then excluded if they were <10 kb in size, covered by <10 probes or covered by <1 probe per 20 kb. PLINK^25^ was used to exclude CNVs that overlapped low copy repeats by more than 50% of their length or had a sample frequency of >1%. This study examined 11 specific CNVs robustly associated with schizophrenia (*P*<4.1 × 10^−4^) in the largest assessment to date of CNVs previously implicated in schizophrenia (Supplementary Table 1).^5^ Of the complete sample passing quality control for both CNV and SNP analysis, 145 cases and 33 controls had a schizophrenia-associated CNV.

### Polygenic RPS analyses

The RPS methodology follows the procedure that has been widely adopted since its first description by the International Schizophrenia Consortium.^29^ We used a training data set as provided by the Schizophrenia Working Group of the Psychiatric Genetics Consortium to identify the set of score alleles from which we derived RPS for each member of the (test) CLOZUK and control samples. The training set, as confirmed by analysis of individual genotype data by the PGC, contains no overlap with the test data set. For those seeking to replicate the findings in samples independent of the PGC, note the full set of SNPs required for RPS is available at https://www.med.unc.edu/pgc/downloads. Using the ‘score' command in PLINK,^25^ scores were calculated by summing the number of susceptibility alleles of each SNP weighted by the logarithm of the SNP OR. We used 10 progressive *P*-value thresholds (*P*_T_) to identify score alleles (*P*_T_<1 × 10^−8^, 1 × 10^−6^, 1 × 10^−4^, 1 × 10^−3^, 0.01, 0.05, 0.1, 0.2, 0.5, 1). Logistic regressions tested whether the resulting RPS was associated with phenotype status.

Under the polygenic liability threshold model, the key prediction is that cases with a schizophrenia-associated CNV would have a higher RPS than controls. To test this, we contrasted RPS in the following tests (see also the section on power below):

1. Schizophrenia cases without a schizophrenia-associated CNV vs controls. This test is a positive control for the ability of RPS to predict affected status generally in our sample.
2. Schizophrenia cases with a schizophrenia-associated CNV vs controls. This is the key test of the primary hypothesis.
3. Schizophrenia cases with a schizophrenia-associated CNV vs controls with a schizophrenia-associated CNV. This is an additional (though less-well-powered) test of the primary hypothesis.

Given the above primary tests supported the polygenic liability threshold model, we undertook a number of secondary tests. We postulated that people carrying CNVs would require a lower burden of common alleles to become affected than those without a CNV; that within cases who are CNV carriers, those with large effect CNVs (defined here as above the median OR, that is, >7) would have lower RPS than those with CNVs with lower effect sizes, but that each subgroup would still have higher RPS than controls. Effect sizes for CNVs were taken from Rees *et al.*^5^ To test the above we contrasted RPS in:

1. Schizophrenia cases with a schizophrenia-associated CNV vs schizophrenia cases without a schizophrenia-associated CNV;
2. Schizophrenia cases with a schizophrenia-associated CNV with an OR greater than 7 vs schizophrenia cases with a schizophrenia-associated CNV with an OR less than 7;
3. Schizophrenia cases with a schizophrenia-associated CNV with an OR greater than 7 vs controls without a schizophrenia-associated CNV;
4. Schizophrenia cases with a schizophrenia-associated CNV with an OR less than 7 vs controls without a schizophrenia-associated CNV; and
5. Ordinal logistic regression by CNV OR, which tests for a relationship between RPS and ranked ORs of the CNVs.

The within-case tests are more limited in power (see the section on power).

Each analysis included six population covariates. We calculated the proportion of variance explained (Nagelkerke's *R*^2^) by subtraction of a full model (covariates+RPS) score from a reduced model (covariates only).

### Polygenic scores analysis power calculation

We performed power calculations for RPS using the methodology and R script available from Dudbridge.^30^ The calculation takes into account factors including discovery and training sample size, number of SNPs used, case−control ratio and proportion of total variance that is explained by common genetic effects (*h*^2^_SNP_) in both the discovery and training data sets. The PGC-SCZ sample size without CLOZUK was 67 992, with a sampling proportion of cases of 0.422. The CLOZUK set of cases without a pathogenic CNV plus all controls was 11 283 with a sampling proportion of cases of 0.468. Life-time risk for schizophrenia was set to 0.01,^31^ with the proportion of total variance that is explained by common genetic effects (*h*^2^_SNP_) set to 0.25 (ref. 7); 96 874 SNPs were used for *P*_T_ <0.5, the threshold we use to illustrate the power calculation.

We calculated the value of *h*^2^_SNP_ for which we had 80% power. For the analysis of schizophrenia cases with a schizophrenia-associated CNV vs controls, the sample size restricted to cases with a pathogenic CNV plus all controls was 6148, with a sampling proportion of cases of 0.023. Prevalence of individuals with schizophrenia carrying a known pathogenic CNV was set to 2.70 × 10^−4^ (rate in the general population of people with schizophrenia with one of the known pathogenic CNVs). For the analysis of schizophrenia cases with a schizophrenia-associated CNV vs controls with a schizophrenia-associated CNV, the CLOZUK sample size of CNV carriers was 178, with a sampling proportion of cases with a pathogenic CNV to controls with a pathogenic CNV of 0.78, and the prevalence of schizophrenia among individuals carrying a known pathogenic CNV being set to 0.0651.^14^ For schizophrenia cases with a schizophrenia-associated CNV vs schizophrenia cases without a schizophrenia-associated CNV, CLOZUK sample size was 5423 with a sampling proportion of cases with and without a pathogenic CNV of 0.027.

## Results

### Polygenic RPS analyses

**Figure 1:**
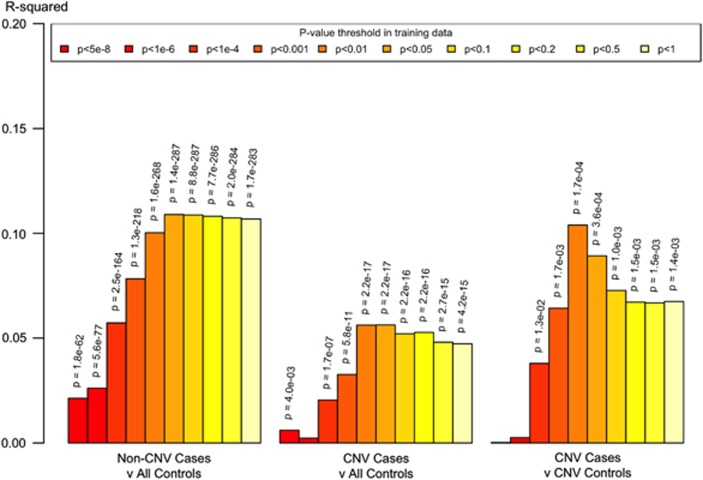
Proportion of variance in schizophrenia in CLOZUK explained by risk profile scores. *R*^2^ is Nagelkerke's *R*^2^ obtained by subtracting the *R*^2^ of the full model (covariates+RPS) from the *R*^2^of a reduced model (covariates only). Ten different training *P*-value thresholds (*P*_T_) for selecting risk alleles are denoted by the colour of each bar (legend above plot). Two-sided *P*-values for evidence at *P*<0.05 are displayed. For each analysis of A vs B, the first sample was coded as 1 and the second as 0 in the logistic regression. CNV, copy number variant; RPS, risk profile score.

Schizophrenia cases without a schizophrenia-associated CNV had a significantly higher RPS for schizophrenia compared with controls (*P*_T_=0.05, *R*^2^=0.109, *P*=1.43 × 10^−287^; Figure 1). In the tests of the primary hypothesis, cases with a schizophrenia-associated CNV had a significantly higher RPS than controls (*P*_T_=0.05, *R*^2^=0.056, *P*=2.25 × 10^−17^); indeed, even among the small number of CNV carriers, RPS significantly distinguished between those with and without schizophrenia (*P*_T_=0.05, *R*^2^=0.089, *P*=3.58 × 10^−4^), cases having a higher RPS than controls.

### Polygenic RPS analysis by CNV OR

**Figure 2:**
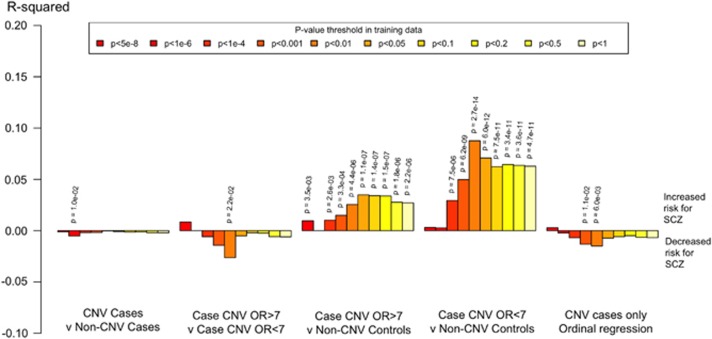
Proportion of variance in schizophrenia in CLOZUK explained by risk profile scores by CNV OR. *R*^2^ is Nagelkerke's *R*^2^obtained by subtracting the *R*^2^ of the full model (covariates+RPS) from the *R*^2^ of a reduced model (covariates only). Ten different training *P*-value thresholds (*P*_T_) for selecting risk alleles are denoted by the colour of each bar (legend above plot). Two-sided *P*-values for evidence at *P*<0.05 are displayed. For each analysis of A vs B, the first sample was coded as 1 and the second as 0 in the logistic regression. *R*^2^ values above 0 symbolize that case status is associated with increased risk for schizophrenia and *R*^2^ values below 0 symbolize that case status is associated with decreased risk for schizophrenia. CNV, copy number variant; OR, odds ratio; RPS, risk profile score.

Among the cases, schizophrenia RPS was not significantly different between those with and without a schizophrenia-associated CNV (*P*_T_=0.05, *R*^2^<0.0001, *P*=0.334); Figure 2 and Supplementary Figures 1 and 2. We found evidence that common alleles contribute to risk in carriers of high OR CNVs and to risk in those with low OR CNVs (high OR vs controls; *P*_T_=0.05, *R*^2^=0.0341, *P*=1.10 × 10^−7^: low OR vs controls; *P*_T_=0.05, *R*^2^=0.0708, *P*=5.99 × 10^−12^) (Figure 2). As expected under a liability threshold model, at most *P*_T_ thresholds, schizophrenia cases with a high OR (OR>7) CNV had a lower RPS for schizophrenia compared both with cases without a known schizophrenia-associated CNV and with cases with a lower OR (OR<7) CNV (Figure 2). However, the results were only nominally significant at a subset of the *P*_T_ values; hence, although the trends are in the direction predicted by the hypotheses, we do not consider these secondary tests as robust. We found no consistent evidence for a difference in RPS between schizophrenia cases with a low OR (OR<7) CNV compared with cases without a schizophrenia-associated CNV.

### Polygenic RPS analysis power calculation

We had complete power to detect an RPS difference between cases without CNVs and controls. We had 80% power to detect an RPS difference between cases with a schizophrenia-associated CNV vs controls even if the *h*^2^_SNP_ (proportion of total phenotypic variance that is explained by common genetic effects) for schizophrenia in CNV carriers is as low as 0.0175. In our analysis of cases with a schizophrenia-associated CNV vs controls with a schizophrenia-associated CNV, we had 80% power to detect an RPS difference if the *h*^2^_SNP_ for schizophrenia to CNV carriers was 0.25, the same as it is for schizophrenia in general. For the secondary analysis, we had 80% power to detect an RPS difference between cases with schizophrenia-associated CNV and cases without a schizophrenia-associated CNV, even if the *difference* in the contribution of *h*^2^_SNP_ to each group was as little as 0.043. We therefore conclude our analyses are well powered, although we cannot exclude small reductions in the burden of common risk alleles in cases that are CNV carriers compared with cases that are not.

## Discussion

The primary goal of this study was to test the hypothesis that within schizophrenia, individuals defined by known pathogenic mutations are completely distinct from the wider population of people with the disorder with respect to their genetic aetiology. Our results do not lend support to the extreme heterogeneity hypothesis. Instead, we found that common risk alleles contribute to the disorder in those who also carry a defined relatively high-penetrance mutation. This was also true for CNVs whose effect size is at the larger end of the spectrum (OR>7), and even the 3q29 and 22q11 deletions that have exceptional effect sizes still had elevated burdens of common alleles (see Supplementary Figures 3 and 4), although we did not have sufficient power to evaluate RPS for individual CNVs. Since the common risk alleles are derived from studies composed almost entirely of those without pathogenic CNVs, this must suggest that shared risk variants operate in those with and without a CNV. In so far as risk alleles must ultimately reflect biological perturbations that result in pathophysiological processes, it follows that those with distinct, defined rare mutations do not form subgroups with a circumscribed pathophysiology.

Our finding is relevant for pathophysiological models of schizophrenia. If an extreme heterogeneity model operates whereby despite similar symptoms, there exists a myriad of sub-disorders defined by non-overlapping aetiologies and distinct pathophysiologies, it might also be the case that studies aiming to model high-penetrance mutations in model systems would have no, or very limited applicability, for the disorder more widely. We do not interpret our findings as suggesting that schizophrenia is a homogeneous disorder where every individual has an identical set of risk alleles and pathophysiology. Rather, we suggest that people with the disorder, including carriers of mutations of large effect, have overlapping pathophysiologies and that these are not demarcated by a single mutation. Inevitably, the degree to which risk alleles and pathophysiological processes are shared between pairs of individuals will vary, but that they do overlap, albeit to a greater or lesser extent, makes it likely that mutations of large effect will have their effects mediated by processes that are relevant to a proportion of people with the disorder who do not carry that mutation.

A second important observation, albeit one that almost inevitably flows from the primary observation, is that among CNV carriers the RPS statistically differentiates between those who do and do not develop schizophrenia. Although the variance currently explained maximizes at around 0.1 (Figure 1), this offers the possibility that as more of common genetic variance for schizophrenia is captured, RPS scoring might make a contribution to predicting the adult psychiatric phenotypic consequences of carrying large-effect, but incompletely penetrant alleles.

As is the case for predicting affected status more widely, it seems unlikely that RPS alone will offer sufficient discriminatory power.^32^ However, one might envisage that it could do so in the future when combined with additional endophenotypes (used here to denote heritable measures like RPS and index genetic liability) or with measures (for example, brain imaging, childhood cognition and psychosocial development) that do not necessarily meet the criteria for endophenotypes (see GotteFsman and Gould^33^ for full discussion) but may nevertheless index increased risk of the disorder. A separate question that will require the exploration of larger GWAS data sets of those disorders is whether RPS might also be used to discriminate between different childhood outcomes, for example being developmentally unimpaired vs having ADHD, and/or ID, and/or ASD, or indeed some of the other adult outcomes that are associated with one or more of the CNVs we have investigated such as epilepsy and obesity.^10^ This will require the exploration of larger GWAS data sets of those disorders, but it seems reasonable to propose that factoring in RPS (and other risk factors) for those disorders might similarly begin to provide predictive power.

## Conclusions

Individuals with schizophrenia harbouring a schizophrenia-associated CNV also have a significant predisposition arising from common risk alleles that influence risk in the wider population of people with the disorder. Our results offer support for a polygenic threshold model rather than an extreme heterogeneity model of schizophrenia, even within CNV carriers. The additional contribution from common alleles may in part explain why some individuals develop schizophrenia rather than another disorder linked to the same CNV.
