# Supplementary material for: Common alleles contribute to schizophrenia in CNV carriers
Source: Mol Psychiatry. 2015 Sep 22;21(8):1085–9. doi: 10.1038/mp.2015.143 (PMC4960448; doi:10.1038/mp.2015.143)
Supplement: Supplementary Information [file mp2015143x1.doc]

Supplementary Materials

# Title

Common alleles contribute to schizophrenia in CNV carriers

# Authors

Katherine E. Tansey^1^, Elliott Rees^1^, David E Linden^1^, Stephen Ripke^2^, Kimberly D. Chambert^2^, Jennifer L. Moran^2^, Steven A. McCarroll^2,3^, Peter Holmans^1^, George Kirov^1^, James Walters^1^, Michael J. Owen^1^, Michael C. O’Donovan^1^

**Supplementary Table 1**: Name, location for the schizophrenia associated CNVs used in this paper.

| CNV Locus | Position in Mb | Case  (n=5 423) | | Controls  (n=6 005) | | Odds Ratio (OR)  (95% CI) | p-value |
| --- | --- | --- | --- | --- | --- | --- | --- |
|  |  | n | Freq, % | n | Freq, % |  |  |
| 1q21.1 del | chr1:146,57-147,39 | 11 | 0.20 | 1 | 0.02 | 12.20 (1.57-94.56) | 0.017 |
| 1q21.1 dup | chr1:146,57-147,39 | 7 | 0.13 | 2 | 0.03 | 3.88 (0.81-18.68) | 0.091 |
| NRXN1 del | chr2:50,15-51,26 | 9 | 0.17 | 3 | 0.05 | 3.33 (0.90-12.29) | 0.072 |
| 3q29 del | chr3:195,73-197,34 | 4 | 0.07 | 0 | 0 | 9.97 (0.54-185.29) | 0.123 |
| WBS dup | chr7:72,74-74,14 | 2 | 0.04 | 0 | 0 | 5.54 (0.27-115.40) | 0.269 |
| 15q11.2 del | chr15:22,80-23,09 | 38 | 0.70 | 17 | 0.28 | 1.83 (1.00-3.34) | 0.050 |
| AS/PWS dup | chr15:24,82-28,43 | 7 | 0.13 | 0 | 0 | 16.63 (0.95-291.28) | 0.054 |
| 15q13.3 del | chr15:31,13-32,48 | 2 | 0.04 | 0 | 0 | 5.54 (0.27-115.40) | 0.269 |
| 16p13.11 dup | chr16:15,51-16,30 | 22 | 0.41 | 8 | 0.13 | 3.05 (1.36-6.86) | 0.007 |
| 16p11.2 dup | chr16:29,64-30,20 | 25 | 0.46 | 2 | 0.03 | 13.90 (3.29-58.72) | 3.00E-04 |
| DiGeorge/VCFS del | chr22:19,02-20,26 | 16 | 0.30 | 0 | 0 | 36.65 (2.20-611.05) | 0.012 |
| Total | | 145 | 2.67 | 33 | 0.55 | 4.97 (3.40-7.27) | 1.27E-16 |


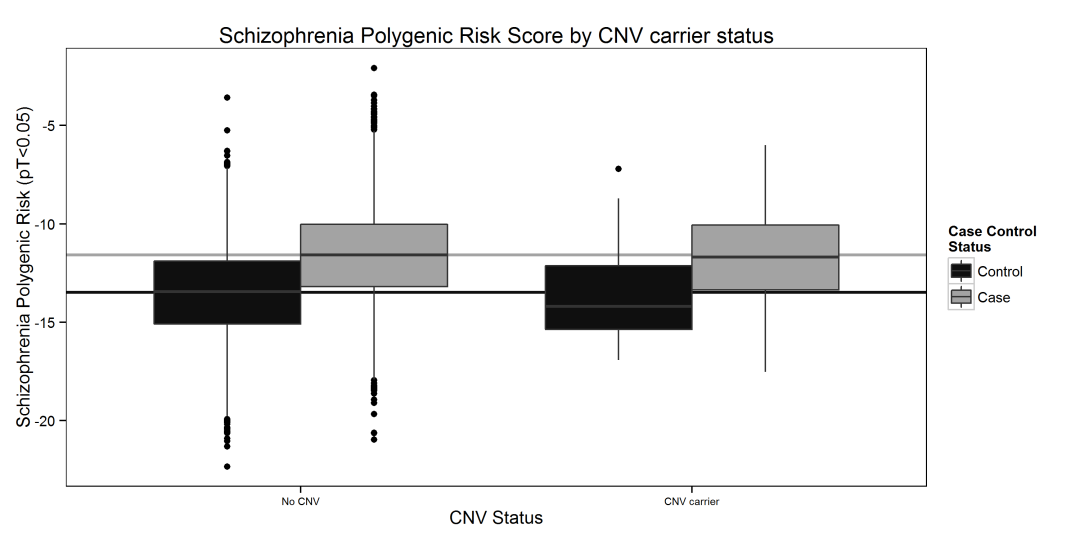


**Supplementary Figure 1: Boxplot of polygenic risk score for schizophrenia by CNV status.** RPS plotted is P_T_<0.05. On the left are individuals without a schizophrenia associated CNV, and on the right are individuals with a schizophrenia associated CNV. Grey is schizophrenia cases and black is controls. Grey horizontal line is the mean for schizophrenia cases without a schizophrenia associated CNV. Black horizontal line is the mean for controls without a schizophrenia associated CNV. Edges of the boxes are the first and third quartiles respectively, with the band displaying the median.


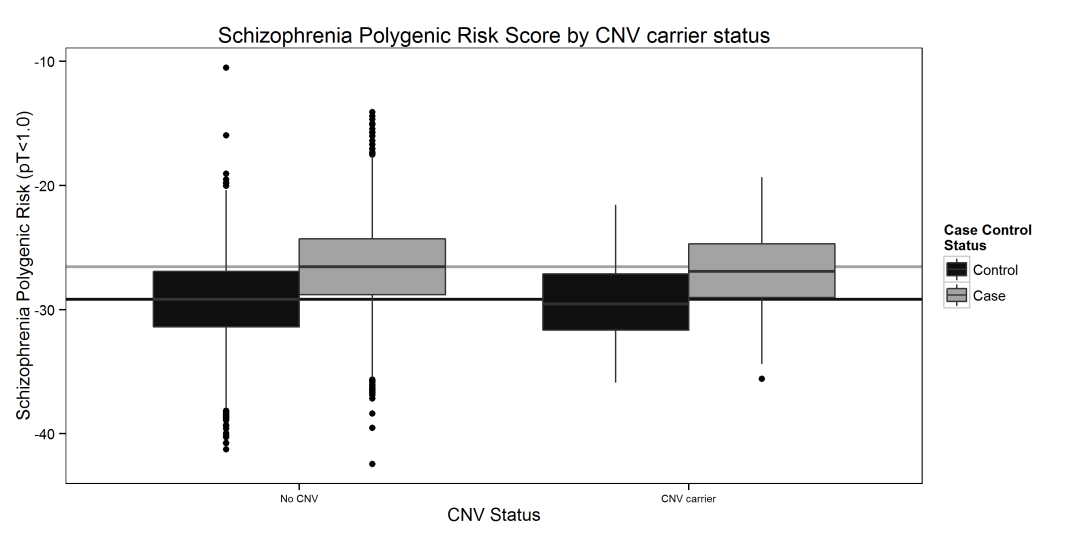


**Supplementary Figure 2: Boxplot of polygenic risk score for schizophrenia by CNV status.** RPS plotted is P_T_<1. On the left are individuals without a schizophrenia associated CNV, and on the right are individuals with a schizophrenia associated CNV. Grey are schizophrenia cases and black are controls. Grey horizontal line is the mean for schizophrenia cases without a schizophrenia associated CNV. Black horizontal line is the mean for controls without a schizophrenia associated CNV. Edges of the boxes are the first and third quartiles respectively, with the band displaying the median.


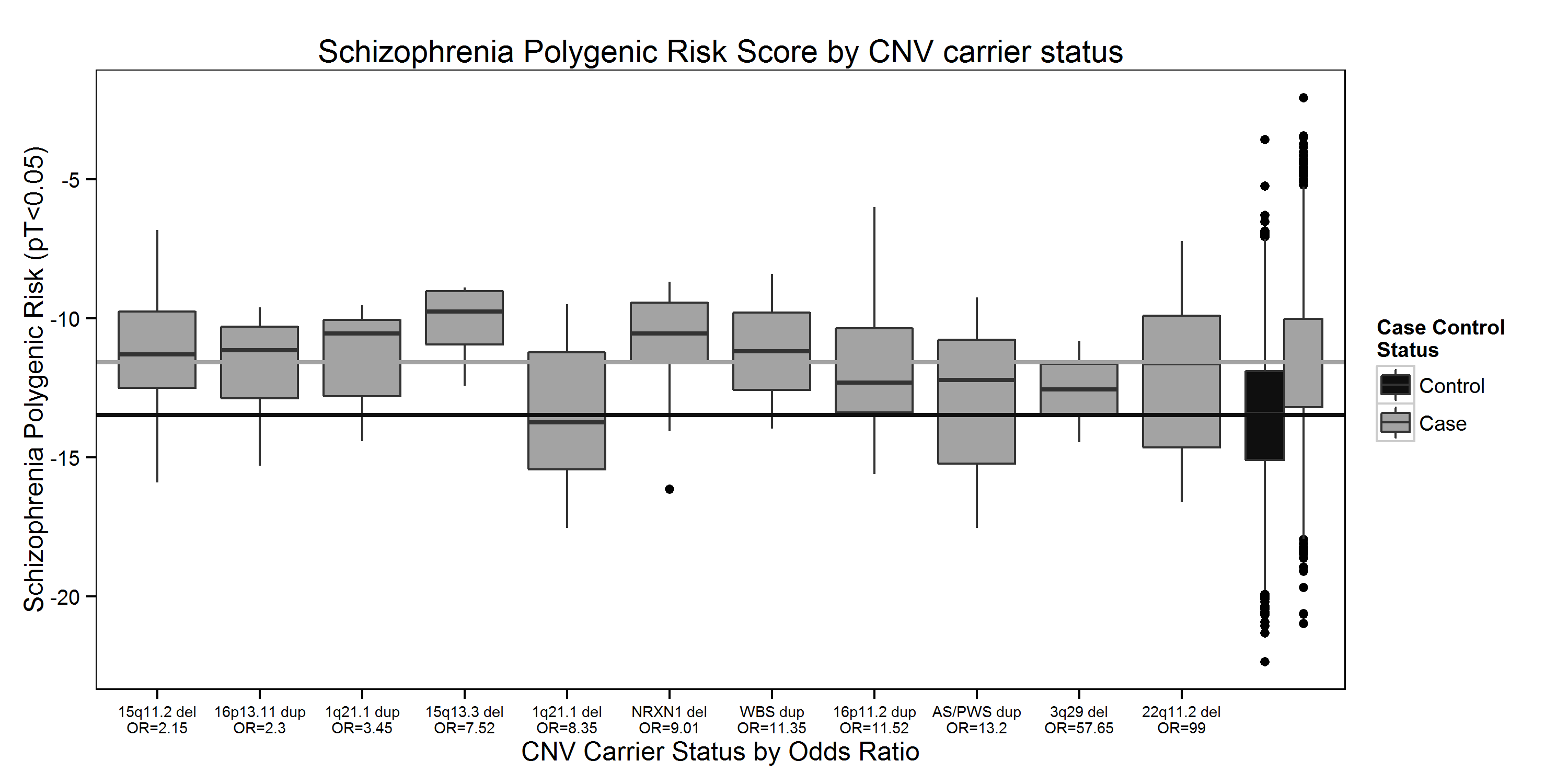


**Supplementary Figure 3: Boxplot of polygenic risk score for schizophrenia by CNV odds ratio (OR).** RPS plotted is P_T_<0.05. OR (as defined by Rees et al[^1^](#_ENREF_1)) increase from left to right with the last boxplot representing individuals without CNVs. Grey are schizophrenia cases and black are controls. Grey horizontal line is the mean for schizophrenia cases without a schizophrenia associated CNV. Black horizontal line is the mean for controls without a schizophrenia associated CNV. Edges of the boxes are the first and third quartiles respectively, with the band displaying the median.


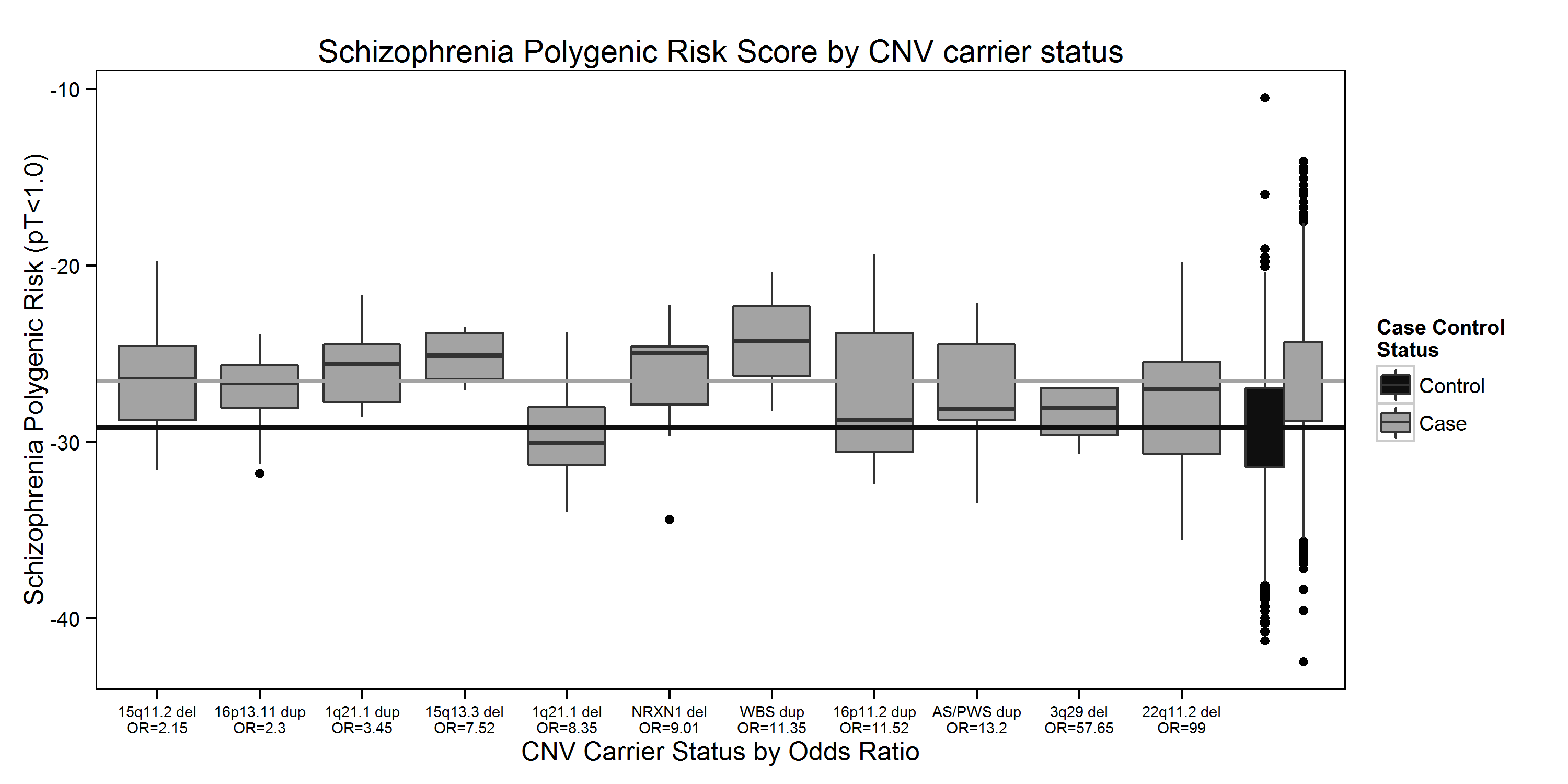


**Supplementary Figure 4: Boxplot of polygenic risk score for schizophrenia by CNV odds ratio (OR).** RPS plotted is P_T_<1.0. OR (as defined by Rees et al[^1^](#_ENREF_1)) increase from left to right with the last boxplot representing individuals without CNVs. Grey are schizophrenia cases and black are controls. Grey horizontal line is the mean for schizophrenia cases without a schizophrenia associated CNV. Black horizontal line is the mean for controls without a schizophrenia associated CNV. Edges of the boxes are the first and third quartiles respectively, with the band displaying the median.

# References:

1. Rees E, Walters JT, Georgieva L, Isles AR, Chambert KD, Richards AL *et al.* Analysis of copy number variations at 15 schizophrenia-associated loci. *The British journal of psychiatry : the journal of mental science* 2014; **204**(2)**:** 108-114.
